# Collaborative Wayfinding: A Critical Review

**DOI:** 10.1002/wcs.70040

**Published:** 2026-09-21

**Authors:** Pablo Fernandez Velasco, John Sutton

**Affiliations:** ^1^ Centre for the Sciences of Place and Memory University of Stirling Stirling UK

## Abstract

Though people can navigate alone, collaborative wayfinding is ubiquitous in human life. Use of GPS devices does not inevitably make it easy to navigate together: conflict in collaborative wayfinding is familiar to most of us, as is failure at it. Yet we know little about how people find their way together. What mechanisms and conditions support effective group dynamics and performance? What difference does a shared history make in grounding communication and decision‐making in wayfinding? Only in the last few years have these questions started to be addressed, but recent developments have come from separate disciplines in a disparate way, both methodologically and theoretically. We critically review recent experimental work. We then draw lessons from five mature fields in the study of collaborative cognition: social ontology, distributed cognition, transactive memory, group problem‐solving, and collaborative recall. Sometimes, navigating together effectively depends on jointly remembering spatial information encoded together during earlier joint action. We suggest tractable designs to identify shared spatial knowledge and assess collaborative process gains and losses, alongside better methods to tap the microprocesses of embodied interaction in small groups remembering routes together. We suggest ways to test for emergence in collaborative wayfinding, and to assess varying divisions of cognitive labor in groups with matching or competing spatial capacities or strategies.

## Graphical abstract

Collaborative wayfinding is ubiquitous in human life. Identifying research lessons from more established fields in collaborative cognition research, we suggest ways to test for emergence in collaborative wayfinding, and to assess varying divisions of cognitive labor in groups.

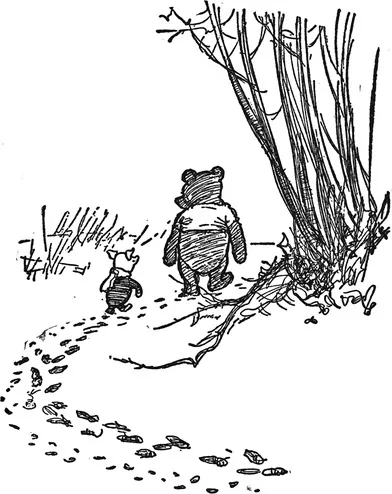

## 1 Introduction

If you are a participant in a typical wayfinding experiment, you are on your own. Maybe you are shown a map of an environment and then asked to navigate it. Or perhaps you get to explore the environment, and then you are asked to take a shortcut. But you cannot ask the experimenter for directions or follow someone who looks like they know where they are going, and there is certainly no phone‐a‐friend option. Outside the confines of the lab, in contrast, wayfinding is a social affair. You ask for directions, or you let a local friend guide you around. Or perhaps you are using a map or satellite navigation, and you are arguing vehemently with your companions about how to interpret the map or how to make sense of the instruction “keep left and stay on the right lane.” Walking together is so ubiquitous that it has become a paradigm case of joint action for philosophers (Gilbert 1990; Fernández Velasco 2024; Bloom‐Christen 2026). And in anthropology, there are many reports of how groups in disparate cultures all around the world collaborate to find their way (see Box 1). In experimental cognitive science, however, it is only in recent years that a focus on the social and collective dimensions of wayfinding has emerged alongside research on the individual.

This recent progress has been hampered by a lack of integration. There have been substantial theoretical, methodological, and empirical developments, but they come from a plethora of disciplines. In this perspective, we draw lessons for the emergent field of collaborative wayfinding from five more established areas of research in collaborative cognition—group problem‐solving, social ontology, distributed cognition, transactive memory, and collaborative recall. As a result, we outline theoretical and methodological considerations for the field. We believe that the resulting framework can provide important common ground for the nascent effort to understand how we find our way together.

## 2 An Interdisciplinary Overview

Wayfinding involves navigating to a goal location, typically in a known environment (Hegarty et al. 2023). Two important dimensions when theorizing about collaborative wayfinding are synchrony and interaction strength (Dalton et al. 2019). Collaborative wayfinding can be synchronous (when a group is co‐present in time and space) or asynchronous (e.g., when one member of the group leaves cues for another member). And the interaction can be weak (e.g., when a group follows a leader) or strong (e.g., when there is active communication among members about different wayfinding decisions). There is a well‐established line of research on the weak synchronous wayfinding of crowd dynamics (Helbing et al. 2005; Moussaid et al. 2009; Kinateder and Warren 2021). There is also substantial work from psychology and linguistics on communication during wayfinding, such as giving route directions (Wunderlich 1982; Denis et al. 1999; Kataoka 2013; Andrews‐Todd and Rapp 2024).

**FIGURE 1:**
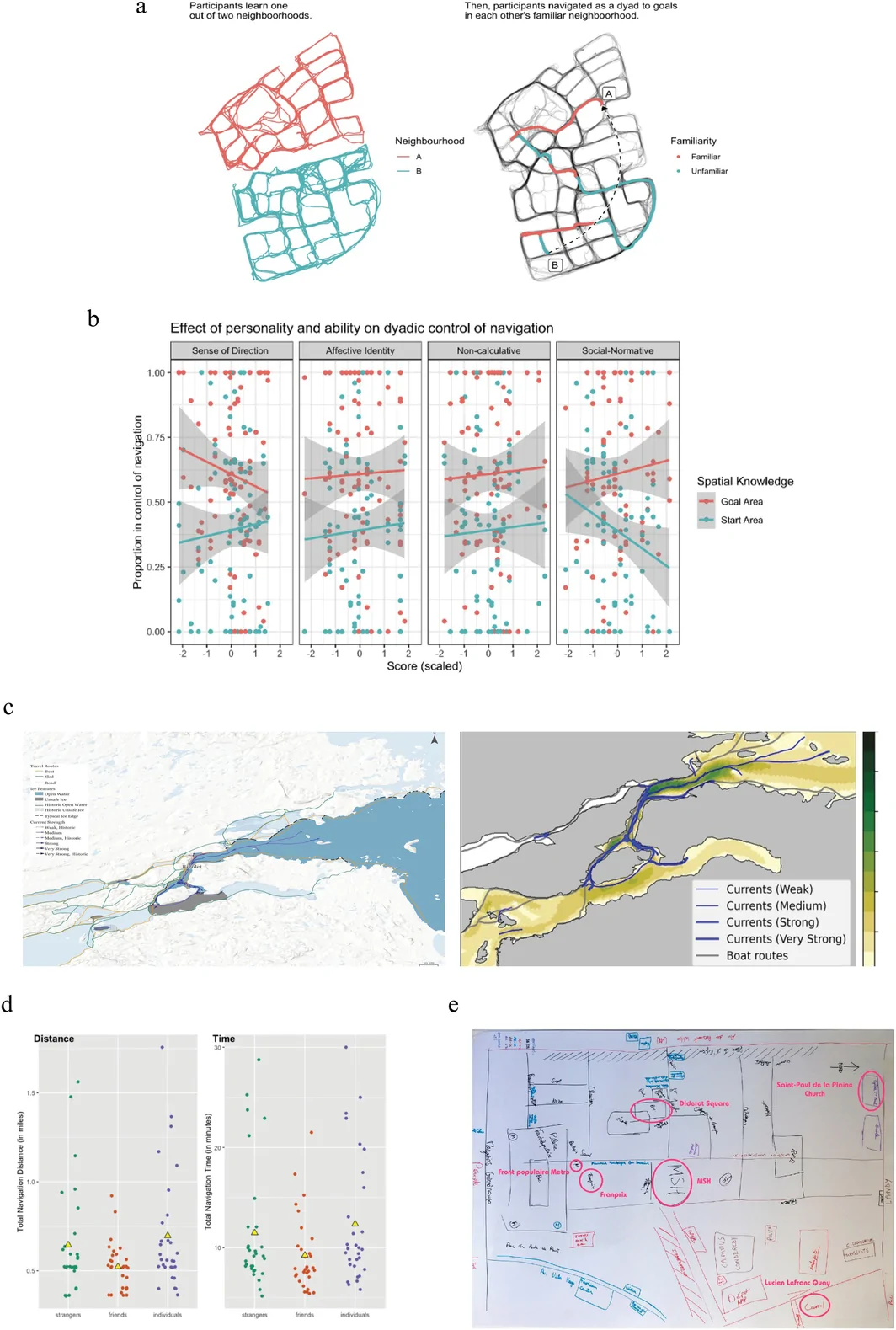
Collaborative wayfinding in different contexts. (a) In this study, two groups explore two different neighborhoods, and then a dyad with a participant from each group has to navigate from one neighborhood to the other (Mavros et al. 2022). The image shows maps of key behavioral data from wayfinding during the individual learning phase (left) and the collaborative wayfinding phase (right). During the wayfinding phase, participants alternate between being in a familiar environment. We can see that participant A (unfamiliar with neighborhood B) leads wayfinding at first, and then hands control over to participant B. This study is an excellent example of synchronous wayfinding. (b) Results from the same study, showing the relationships between each participant's characteristics and the proportion in control of the wayfinding in each trial. This shows the dynamic interaction dimension of wayfinding. (c) Collaborative mapping between oceanographers and Inuit people in the Rigolet community (Bishop et al. 2022). Left panel is the Rigolet community mapped trails and oceanographic features. Right panel shows ocean current features mapped by Rigolet residents (blue lines) and predictions of current speeds from the computational model (yellow‐green shading). This shows how collaboration can operate even in asynchronous interactions. (d) Wayfinding performance of individuals, dyads formed by two friends and dyads formed by two strangers (Bae et al. 2024). Jitter plot of total distance and time across the three studies. Triangle marker represents mean values. The study shows collective inhibition in wayfinding. Interestingly, although they found the friend dyads proposed more unique route plans than stranger dyads, friend dyads were less efficient overall in reaching the correct destination. (e) Collaborative map completed by participants who had explored a neighborhood alone and unaided (Quesnot and Guelton 2023). Global landmarks highlighted in pink.

Experimental psychologists have recently started to contrast cases of wayfinding that vary along the synchrony and interaction dimensions (Figure 1). Bönsch et al. (2024) explored three different conditions in a virtual, sparsely populated, urban environment: unsupported wayfinding, strong social wayfinding (a virtual supporter guide), and weak social wayfinding (flows of virtual agents that subtly influence the participants' wayfinding decisions through their movement). They found that strong social wayfinding supported knowledge acquisition and was less cognitively demanding, while, unsurprisingly, weak social wayfinding worked better for participants who prioritized autonomy. Another study (Yassin et al. 2021) iterated various configurations in a wayfinding task: individuals versus pairs, and copresence of either player‐controlled or nonplayer‐controlled characters. They found that the impact of copresence varies depending on the social scenario. They also found that copresence changed how participants experienced wayfinding aids (e.g., signs and lighting). Finally, they found that social factors such as role‐taking and empowerment drove the difference between the behavior of people navigating individually or in dyads. Other likely important factors in collaborative wayfinding are interpersonal dynamics (Brunyé et al. 2023), social influence (Silva et al. 2015), gender (Kallioniemi et al. 2015), and age (Rybarczyk et al. 2023). Variation across these factors, and across the two different dimensions of collaborative wayfinding, highlights the many elements in a cognitive ecosystem that affect how collaborative wayfinding unfolds (Fernández Velasco and Sutton 2025).

BOX 1 **Anthropological Accounts of Collaborative Wayfinding.**

Several ethnographic accounts from anthropology offer interesting insights into group navigation (Fernández Velasco and Spiers 2024). Every year, Inuit expert hunters break trails across the sea ice to make them visible for other travelers (Aporta 2004). Trail breaking is an important aspect of traditional wayfinding among the Yellowknives Dene First Nation people, involving division of labor, with some hunters breaking the trail at night and the rest of the group following the trail in the morning (Cannon et al. 2022). Collaborative wayfinding involves coordination, sometimes over long distances: here signals play an important role, as when sailors determine the location of a ship in the fog from the sound of its whistle (Lee et al. 2021). Warburton Ranges people of the desert of Western Australia, for example, make a fire and use smoke to convey information about a location, a technique that can indicate, for example, that there is a water source next to the origin of the smoke (Gould 1980). Anthropology research can tell us a lot about how group composition changes wayfinding behavior in real‐world contexts. For instance, a study in Congo compared foraging trips of BaYaka mothers going with children and without children in terms of length, distance, energy expenditure, and food return (Visine et al. 2024). Anthropological literature also points to the importance of emotion, and how key affective cultural practices often are for upholding navigational culture. In the Inuit Wainwright community, shaming worked as a teaching device. Travelers who got lost would be collectively shamed, as a form of encouraging the learning of navigational culture (Sonnenfeld 2002). Finally, a recent trend in anthropology is multi‐species ethnography (Hartigan Jr 2021), and wayfinding sometimes involves interaction with non‐human animals. For Inuit communities, animal behavior can serve to predict changes in wind patterns (McMahan et al. 2022). And among the Eveny, the reindeer largely determine the herder's trajectory (Vitebsky and Alekseyev 2020).

The simplest example of strong synchronous wayfinding is wayfinding in pairs (Ishikawa 2023), when the relationship between group members can be studied and manipulated (Forlizzi et al. 2010). One study compared dyads who were already friends with dyads formed by two strangers (Bae et al. 2024). The results are complex and interesting. The friend dyads spent about as much time as strangers prospectively planning their routes but proposed more unique route plans and adhered more closely to the planned route in the situated navigation task. However, fewer friend dyads were successful overall in reaching the correct destination. Social familiarity within the dyad may have lent friends more wayfinding confidence, which could both have led them to be less flexible in adapting plans in the situated context due to over‐confidence. Another important element is uncertainty about spatial information. When members of a dyad have explored different neighborhoods, navigational control shifts according to each member's familiarity with the surrounding environment (Mavros et al. 2022). Dyadic interaction can involve flat decision‐making or a division into social roles, such as leader and follower. These social roles can emerge spontaneously through interaction (Bae and Montello 2019) or by design (Forlizzi et al. 2010; Gridling et al. 2012; Bacim et al. 2012; Bryden et al. 2014). Another division into social roles is a division of labor according to wayfinding subtasks (such as route following versus path integration) or to different search areas. In collaborative search, there is a trade‐off between a spatial division‐of‐labor strategy (Malcolmson et al. 2007; Brennan et al. 2008) and tracking the partner's perspective to establish common ground (Schwarzkopf et al. 2017).

Ethnographic work has also addressed collaborative wayfinding (Hutchins 1995). Almeida and Tidal (2022) conducted a qualitative study in a library setting, and their data show how power dynamics and communication patterns shape collaborative wayfinding behavior. Another study explores how firefighters navigate response areas (McWhorter et al. 2025). The focus is on learning and training, which is an important aspect of collaborative wayfinding, especially when we look at its widely distributed, asynchronous elements. One of the emergent themes was group work, which involved teaching while driving around, informal discussions, mentoring, teamwork, as well as practical learning such as drills. They also examined how routing decisions were made during calls based on social role and context. Finally, they found that firefighters who work as part of a group use their personal navigation aids less than those firefighters who work solo, showing how technology use interacts with other elements of collaborative wayfinding. There is also a relevant body of work about the role of cognitive artifacts in collaboration, such as maps (Morrison et al. 2011; Quesnot and Guelton 2023) or Global Navigation Satellite Systems (Reilly et al. 2009; McCullough and Collins 2019). For instance, looking at oceanographers and Inuit people working together to identify paths and safe areas for travel shows how cognitive artifacts such as maps allow for the flux, interpretation, and recontextualization of knowledge and data across cultures involving not only different spatial knowledge but different ways of knowing space (Bishop et al. 2022).

## 3 Wayfinding as Collaborative Cognition

Collaborative wayfinding is one of many cognitive activities that people often do together. The result of such joint action is not always simply the sum of individual contributions. Forms of interactive, socially distributed, or collaborative cognition have been studied in a range of fields. These have sometimes been relatively marginal traditions, given the long‐standing dominance in cognitive science of individualism and internalism, the ideas that the individual agent is psychologically self‐sufficient, and that processes outside the skull and skin are merely cues to or outputs of the real internal mental goings‐on (see critiques by Dingemanse et al. 2023; Spivey 2024). But five such fields, we suggest, can contribute directly to the emerging sciences of collaborative wayfinding: social ontology, distributed cognition, transactive memory, the psychology of group problem‐solving, and collaborative recall (Table 1). We show how methods for finer‐grained process analysis can be extracted from these fields, operating alongside the study of individual navigation and spatial cognition capacities.

**TABLE 1:** Concepts and methods from collaborative cognition research.

Social ontology
Plural subject
Shared intentions as common knowledge plus meshing sub‐plans
We mode of experience
Distributed cognition
Environmental structure and learning trajectories
Cultural practices and technology use
Triangulating cognitive ethnography and experimental psychology
Transactive memory
Focus on social interactions
Systematic divisions of mnemonic labor
Informational flows
Group problem‐solving
Emergence—when the products of group performance differ from the simple addition of individual products
Synergy—when the group performs better than that sum
Key forms of interaction: group composition, coordination, and communication
Collaborative recall
Establish an individual baseline
Performance metrics (e.g., accuracy, see wayfinding studies for reference)
Consider group composition

Conceptual resources for understanding the experiential and intentional dimensions of wayfinding together can be drawn from philosophical work on social ontology (Epstein 2025). Stronger views in this field describe certain groups as forming a “plural subject” in that members pool their wills, recognizing and acting on a structure of mutual commitments and expectations (Gilbert 1989), or as exhibiting genuine forms of agency in being responsive to reasons and tracking the coherence and constraints of representations and decisions over time (List and Pettit 2011). More modest approaches analyze shared intentions in terms of the common knowledge of group members along with appropriately “meshing subplans” on how to think and act together (Bratman 2013): route planning in dyads and small groups often involves such meshing sub‐plans. Phenomenological philosophy adds rich analyses of the ways that acting and thinking in a “we”‐mode within a group transforms experience, highlighting the roles of empathy and interpersonal engagement, shared emotions, and communal bonds (Zahavi 2024). What it is to navigate *togethe*r rather than merely at the same time may involve distinctive cognitive and affective processes theorized in phenomenology and social ontology.

Wayfinding was a key domain from the start in the ‘situated’ or ‘distributed’ movements that, from the 1990s, transformed the cognitive sciences from within. Hutchins (1995) developed the idea that cognition is distributed across brains, bodies, equipment, and environments through ethnographic and historical study of maritime wayfinding in early modern sailing, Pacific navigation, and a US Navy frigate. Otto, the early‐stage Alzheimer's patient who partly offloads his memory system into a notebook, in Clark and Chalmers' (1998) argument for the “extended mind,” was using the external technology to find his way to a museum. Just as “4E cognition”—embodied, embedded, extended, and enactive—has influenced cognitive scientific research on perception and attention, creativity, emotion‐regulation, and remembering (Newen et al. 2018), it expands the units of analysis in wayfinding research to include both navigation technologies and social processes. In strong cases of cognitive interdependence, capacities like spatial cognition are not simply offloaded onto to artifacts or collaborators, but transformed and reorganized across variable and dynamic wayfinding ecologies (Hutchins 2010; Gillett and Heersmink 2019; Mingon et al. 2026). Cognitive ethnography (Hutchins 2025) then tracks both propagated representational states and interaction processes across people, tools, and environments, and can offer field‐based methods to complement the laboratory tasks discussed below.

Transactive memory systems theory (Wegner 1987) highlights specifically *social* interactions at all stages of the processes of remembering information or experiences, among for example intimate couples (Wegner et al. 1985; Harris et al. 2019) or organizational teams (Ren and Argote 2011). A transactive memory system consists of individual memories alongside particular forms of cognitive organization and communication across and between group members. What is remembered can be divided up differentially in systematic divisions of mnemonic labor, as long as an accurate enough shared higher‐level knowledge of who knows what is maintained, and effective enough forms of access or “transactions” allow the group as a whole to access any information as needed, making certain groups capable of “memory feats” beyond the capacity of any individual group member (Wegner 1995; Theiner 2013; Tollefsen et al. 2013). Transactive memory theory thus underlines the cognitive significance of shared history among group members, the centrality of communicative processes, and the possibility of substantial differentiation among individuals within a group with regard to precisely what each contributes to the collaboration. A transactive memory perspective on collaborative wayfinding directs attention to the division of spatial knowledge within wayfinding groups—who knows which routes, neighborhoods, and landmarks—and how the group accesses, coordinates, and deploys such differentiated spatial knowledge under real navigational demands.

Rigorous paradigms in experimental psychology have addressed group problem‐solving, idea generation and “brainstorming,” and other forms of collaborative cognition, developing taxonomies of distinct kinds of task, and concepts and methods for process tracking to detect gains and losses arising from working together (Steiner 1966; Laughlin 2011). When the products of group performance differ from the simple addition of individual products, we may have “emergence” and even (when the group performs better than that sum) “synergy” (Larson Jr 2010; Theiner et al. 2010). Groups often face particularly difficult cognitive tasks, in which relevant information is unevenly distributed among group members (Stasser and Titus 2003), or has to be integrated effectively in the face of social, affective, or personality differences (D'Agostino 2008), a kind of challenge that can have very high stakes in the context of collaborative wayfinding (Ebert and Morreau 2023). Research on collective intelligence (Woolley et al. 2015) and team cognition (Eccles and Tenenbaum 2004; Hall et al. 2018; Cooke et al. 2024) thus addresses group composition, coordination, and communication as key forms of interaction in domains from sport to emergency response. These traditions provide both conceptual vocabulary—emergence, synergy, process gain and loss—and the task‐analytic rigor that collaborative wayfinding research needs, and that the more specific case of collaborative recall has developed furthest.

Experimental psychologists have therefore sought a higher standard for assessing success or process gain from collaboration than has often been deployed in transactive memory research. It is not enough for a group to outperform an average individual: rather, to live up to its cognitive potential and do better than the sum of its parts, the group must remember more (or otherwise better) than the pooled memories of the same number of individuals remembering alone, what is called the “nominal group” (Weldon 2000; Marion and Thorley 2016; Guynn 2024). The surprising but robust result of decades of work on “collaborative recall” is that typically groups fail to meet this criterion: instead, “collaborative inhibition” is standard, when people remember *less* effectively when collaborating in a group than when alone (Harris et al. 2008; Meade et al. 2018). But, inspired by the theoretical approaches sketched above, recent research has convincingly shown that certain kinds of groups, such as subject experts or people with extensive shared history such as some intimate couples, in contrast can achieve “collaborative facilitation,” using verbal and nonverbal communication strategies effectively enough to perform better together than apart (Meade et al. 2009; Harris et al. 2011, 2019; Harris and McIlwain 2026). As one form of emergence or synchrony, collaborative facilitation is a natural focus for research in wayfinding, which can adapt designs and methods from work on collaborative recall. It is likely that in collaborative wayfinding too, the conditions for process gain will be fragile—that while in many cases the individuals in a couple or a group will not navigate more effectively together than they would alone, there will be cases, contexts, and tasks in which they can work together well enough for collaborative success. To illustrate more specifically how research in the parallel case of collaborative recall offers lessons for wayfinding, we outline a research agenda.

## 4 Research Lessons for Collaborative Wayfinding

In addition to cultural and cross‐cultural aspects of navigation (Box 1), and computational models (Box 2), a natural suggestion for collaborative wayfinding research is that a social component could be added to many experiments on navigation, treating a collaborative condition as part of the paradigm rather than an occasional add‐on. For example, studies manipulating self‐reference and familiarity with neighborhoods (Penaud et al. 2022) could test dyads or small groups to see if collaboration affects the performance of those who know an area well differently with regard to performance in remembering specific events and their spatiotemporal contexts. Studies testing flexibility and spatial strategies in individuals (Castilla et al. 2021, 2022) could add collaborative conditions to see how dyads plan and replan routes in light of blockage, compared to individuals, and whether it is better for group members to share strategy preferences or have different preferred strategies (compare He et al. 2015). Another promising step is adapting the dual‐solution paradigm (Marchette et al. 2011): Participants can learn a route through an environment and then face a choice between following the learned route or taking a novel shortcut. Individual differences in place‐based versus response‐based strategy are robust, so dyad can include one strong cognitive mapper and one strong route follower. Does the group adopt one person's strategy, negotiate a hybrid, or does the mismatch impair performance? And moreover, does the alignment of strategy preferences drive inhibition or facilitation? In the study of spatial cognition more broadly, including collaborative conditions where possible is a key methodological goal as we learn more about the pros and cons of different methods in navigation research for tapping individual differences effectively (Newcombe et al. 2023; Yavuz and Spiers 2026), for allowing participants to move freely (Iggena et al. 2023; Hill et al. 2024), and for combining the control offered by laboratory studies with the ecological realism of research in natural settings such as work or urban environments (Vigliocco et al. 2024).

BOX 2 **Computational Models.**

Computational modeling is central to cognitive science (McClelland 2009). Bringing computational modeling to the study of collaborative wayfinding has enormous potential, and a few recent developments can help pave the way. One of the most basic principles of collaborative wayfinding concerns “many wrongs,” or “vox populi” processes, in which accuracy improves simply by pooling individual estimates, because the accuracy of the group average orientation should be greater than the average of individual orientation accuracy (Bergman and Donner 1964; Berdahl et al. 2018). One way to implement the principle is through copy‐cat dynamics (Codling and Bode 2014). The principle has been observed at work in experimental studies of collaborative wayfinding (Faria et al. 2009). Another simple process is leadership, when informed individuals guide naïve individuals. A recent simulation study leverages a non‐local hyperbolic model (i.e., where interactions depend on influences at a distance through attraction and alignment rather than diffusion) to explore how groups with different degrees of leader's certainty fare in various collective navigation conditions (Bernardi and Painter 2025). A more complex process is emergent sensing, when coordinated wayfinding behavior emerges despite group members being unable to navigate on their own (Torney et al. 2009), such as a distributed sensor network, where individuals make scalar measures of a cue, but the group can compare those measures to sense a gradient (Berdahl et al. 2013; Guttal and Couzin 2010; Hein et al. 2015). A promising development lies at the intersection of collective behavior and generative models of individual behavior. A recent model in this area characterizes the relationship between generative model parameters and emergent information‐processing capacities, which are measured by collective information transfer and responsiveness to external perturbations. The idea is that the alternative generative models underlying the behavior of the agents could be estimated from behavioral or neural data, and the resulting social interaction processes would then emerge as those behaviors that minimize surprise, relative to the generative model that best explains the agent's behavior, which provides a clear computational model that can be applied to collaborative wayfinding studies (Heins et al. 2024). Finally, we can consider social metacognition, in which agents evaluate and regulate the cognitive processes of other members of the group. One way to cash out this notion is explicitly, for example, in terms of transactive memory systems, where there is a shared awareness of who knows what information within a group (Peltokorpi 2008; Montello et al. 2023; Maupin et al. 2023). The idea can also operate at the level of implicit metacognition, where cognition is collectively regulated without the need of meta‐representation (Fernández Velasco 2024).

Wayfinding is very heterogeneous (for a taxonomy, see Wiener et al. 2009), but in some circumstances, collaborative wayfinding just *is* collaborative recall. When people have followed a particular route in the past, whether individually or (in the stronger case) together, doing so again together now is remembering collaboratively, where what is remembered together is spatial content of various forms. Variations on experimental design developed in collaborative recall research are thus available for use in studies of wayfinding: encoding can be individual or collaborative, the modes and processes of interaction at retrieval can vary, and the effects of collaboration on subsequent individual memory for the same content can later be tested. People find their way together, on foot or by car for example, under many varying conditions, with different levels of familiarity (with the environment and with each other), time pressure, stress, and so on, and for many different purposes across contexts. Such everyday collaborative experiences should be at the heart of wayfinding research (Goldenberg et al. 2026), not a rare or optional extra. One suggestive study by Quesnot and Guelton (2023) stands out as a promising initial attempt to link wayfinding with collaborative recall, testing individual and group performance on memory for landmarks and distances across groups who had explored a neighborhood alone (with or without mobile mapping support), and had their individual recall pooled as nominal groups, or together. Standard collaborative inhibition effects were found, as is not surprising in groups of strangers remembering previously unfamiliar content. We recommend this study, which has some striking features in seeking to address a number of research questions at once, as one useful starting point for future attempts to integrate wayfinding and remembering as linked forms of collaborative cognition. Wayfinding performance, of course, goes beyond recall. Accordingly, future studies can look at performance metrics such as deviation from the optimal route, pointing accuracy, or errors at decision points, and then consider inhibition‐vs‐facilitation, as well as variations in experimental design, for those performance metrics.

We single out five further lessons from collaborative recall to encourage further research, focusing briefly in turn on issues relating to groups, communication, individual differences, measures and success, and technology. First, different wayfinding contexts involve groups of different sizes, which permit distinctive dynamics and divisions of knowledge and labor and afford different opportunities for communication and interaction. In particular, the role of experience between group members is vital, but is typically not alone sufficient to drive collaborative benefits—what else is required, under certain circumstances, for shared history to make a difference? And how might collaborative processes scale up to larger groups? Second, wayfinding researchers who have typically studied individual cognition can draw on methods for tapping the microprocesses of multimodal communication among group members, where memory performance in groups seems to depend on subtle mechanisms of cross‐cuing and coordination across nonverbal as well as verbal channels (Harris et al. 2011, 2022; Harris and McIlwain 2026). While spatial configuration, eye gaze, and gesture are important here, research can also investigate unique features of human communication. Social signals in collective search behavior can be “conveyed among participants without any verbal communication” (Hoffman et al. 2025, 1), and experimental designs in which talk is banned do permit clearer comparison with navigation processes in non‐human animal groups (Faria et al. 2009). But verbal communication among group members is absolutely pervasive in ordinary wayfinding, arguably transforming the mechanisms and outcomes of interaction, such that specific forms of linguistic alignment support cooperative task performance (Fusaroli et al. 2012).

A natural third area of focus is on how individual differences among group members may influence collaborative wayfinding. Established methods for tapping individual differences in navigation strategy (Hegarty et al. 2023) can fuel investigations into what different capacities individuals bring *to* a group, and what different and potentially complementary contributions individuals make *in* the group process. This connects to enquiry into the nature and role of expertise in collaborative wayfinding, as we ask how expertise in navigation may relate to expertise in collaboration, and tap the slow development of expertise in joint embodied action (Sutton 2024). Fourthly, we know that collaboration has many functions beyond simple instrumental success as measured, for example, by accuracy or speed. Just as we remember past experiences together in order to maintain relationships and enjoy interaction as well as to latch on to the truth, so we explore places together for many different reasons. So, what counts as success in collaborative wayfinding may change across contexts, complicating our decisions about what exactly to measure. Finally, as it becomes increasingly rare for real‐world wayfinding to be done off‐grid and without technological assistance, we want to understand how people use GPS, satnav, and web‐based mapping services collaboratively. Whether we worry about potential negative effects of digital technology on spatial cognition, or want to see how new navigation tools may be deployed by experts or to transform cognitive processes (Topete et al. 2024; Mingon et al. 2026), we need to study the development and use of integrated sociotechnical systems in collaborative wayfinding.

## 5 Conclusion

When we have to find our way, we rarely do so alone. Collaborative wayfinding is ubiquitous, and yet it is only in recent years that research across the cognitive sciences has started to explore this complex phenomenon. Here, we review some of the studies emerging from both experimental and ethnographic literature on collaborative wayfinding, and we contrast them with work in the more established fields of group problem‐solving, social ontology, distributed cognition, transactive memory, and collaborative recall. This move introduces the notions of emergence (when group performance differs from the addition of individual performances) and synergy (when group performance improves the sum of individual performances). It also highlights the experiential dimensions of collaborative cognition, the importance of communicative processes and the differentiation of individuals within a group. Finally, an important effect is collaborative inhibition, when a group as a whole remembers *less* effectively than its members remembering alone, which serves to operationalize the notion of synergy. Wayfinding studies should consider this effect as well as the situations that facilitate the reverse effect, called collaborative facilitation. We identify five lessons from this cross‐field comparison: collaborative wayfinding depends on context and group size, micro‐processes such as cross‐cuing and coordination drive collaborative dynamics, individual differences in cognitive strategies and capacities shape collective performance, collaboration has many functions beyond speed or accuracy, and technology transforms the ways in which cognitive processes are distributed. All of these are important lessons for the interdisciplinary study of collaborative wayfinding.

## Author Contributions

**Pablo Fernandez Velasco:** conceptualization, funding acquisition, writing – original draft, project administration, writing – review and editing, visualization, investigation. **John Sutton:** conceptualization, funding acquisition, writing – original draft, writing – review and editing, project administration, investigation.

## Funding

This research was supported by the Leverhulme Trust International Professorship Award LIP‐2023_002. Pablo Fernandez Velasco's research was also supported by a British Academy Postdoctoral Fellowship (PFSS23\230053).

## Conflicts of Interest

The authors declare no conflicts of interest.

## Data Availability

Data sharing not applicable to this article as no datasets were generated or analysed during the current study.
